# The effects of TGF-β receptor I inhibitors on myofibroblast differentiation and myotube formation

**DOI:** 10.3389/fcell.2025.1636884

**Published:** 2025-07-22

**Authors:** Zhihao Wang, Edwin. M. Ongkosuwito, Johannes W. Von den Hoff, Frank A. D. T. G. Wagener

**Affiliations:** Department of Dentistry, Orthodontics and Craniofacial Biology, Research Institute for Medical Innovation, Radboud University Medical Centre, Nijmegen, Netherlands

**Keywords:** TGF-βRI inhibitors, myotube, myofibroblast, C2C12, fibrosis

## Abstract

**Introduction:**

Fibrosis frequently occurs in muscle wounds, ultimately leading to suboptimal function. This study investigates the effects of TGF-βRI inhibitors AZ12799734, Galunisertib, and SM16, on myofibroblast differentiation and myotube formation.

**Methods:**

Human gingival fibroblasts were treated with TGF-β1 (0, 1, 5, and 10 ng/mL) to induce myofibroblasts. Then, fibroblasts were incubated with TGF-βRI inhibitors (0, 1, 5, 10, and 20 µM) together with 10 ng/mL TGF-β1. Myofibroblast marker expression was assessed using RT-PCR (day 3), while myofibroblast differentiation was analyzed by immunofluorescence staining for α-SMA (day 6). C2C12 myoblasts were also cultured with TGF-βRI inhibitors, and gene expression (day 3) and myotube formation (day 6) were analyzed.

**Results:**

TGF-β1 (10 ng/mL) increased the proportion of myofibroblasts from 9.3% ± 3.5% to 38.1% ± 4.4%, which was reduced by all TGF-βRI inhibitors even at 1 µM [for example, Galunisertib 23.5% ± 2.1% (p < 0.05)]. All inhibitors reduced ACTA2 and COL1A1 gene expression, while only AZ12799734 and SM16 inhibited Ki-67 expression. In C2C12 cultures, AZ12799734 and SM16 reduced the fusion index, whereas Galunisertib did not. Moreover, only Galunisertib increased myotube size from 0.09 ± 0.01 to 0.13 ± 0.01 mm^2^/nucleus (p < 0.05). Galunisertib inhibited MyoD gene expression (at 20 µM), but not MyoG nor MyHC.

**Discussion:**

Galunisertib may have potential for improving muscle wound healing following injury.

## 1 Introduction

Fibrosis occurs when fibroblasts differentiate into myofibroblasts and deposit large amounts of extracellular matrix (ECM) components in the wound following trauma or surgery. In injured muscle tissue, fibrosis also disrupts tissue integrity. Such disruptions can lead to significant functional and aesthetic impairments, burdening patients and the healthcare system (Park et al., 2022). Fibrosis in the soft palate after cleft palate surgery can hinder velopharyngeal function, adversely impacting speech development (Von Den et al., 2019). Consequently, fibrosis after muscle wounding represents a major obstacle to normal muscle regeneration, function, and growth.

Muscle healing is a complex process involving the coordinated action of (myo) fibroblasts and the formation of myofibers (Von Den et al., 2019). Following injury, fibroblasts differentiate into myofibroblasts, depositing extracellular matrix (ECM) components such as collagen. This process can lead to fibrosis, especially in wounds with a large muscle defect. Meanwhile, activated satellite cells (SCs) proliferate, differentiate into myoblasts, and fuse to form new myofibers, a process essential for restoring functional muscle tissue. When fibrosis predominates, it interferes with myofiber formation, thus impeding muscle regeneration and resulting in suboptimal function (Delaney et al., 2017). Currently, the only available antifibrotic drugs are Nintedanib and Pirfenidone, both of which are used for IPF (Z. Wang et al., 2023). Among these, nintedanib, an FDA-approved drug for idiopathic pulmonary fibrosis (IPF), has demonstrated significant antifibrotic effects *in vitro*, *in vivo*, and in clinical settings (Schreurs et al., 2020). However, given the severe side effects of nintedanib, developing a safer alternative is highly desirable (Bendstrup et al., 2019; Cottin et al., 2022). Thus, more specific small molecules may represent a promising approach for treating muscle fibrosis.

Transforming growth factor-beta (TGF-β) is a key factor in fibrosis, well known to promote myofibroblast differentiation, but also inhibit to myofiber formation (Von Den et al., 2019). Numerous studies have demonstrated that TGF-β1 induces myofibroblast differentiation in various fibroblast types, such as gingival, lung, cardiac, and dermal fibroblasts (Meran et al., 2007; Bell et al., 2022; Kottmann et al., 2012; Qu et al., 2019). Conversely, human SCs or mouse C2C12 cells exposed to TGF-β1 exhibit reduced fusion (Gardner et al., 2011; Shi et al., 2021; H. Wang et al., 2018). TGF-β1 binds to TGF-βRII, forming a heteromeric complex with TGF-βRI, mainly of the activin receptor-like kinase 5 (ALK5). Activated ALK5 recruits and phosphorylates Smad2/3, ultimately inducing the transcription of target genes (Hill, 2016). Consequently, targeting the TGF-βRI presents a potential strategy for mitigating fibrosis and enhancing muscle regeneration. Small-molecule TGF-βRI inhibitors such as AZ12799734, Galunisertib, and SM16 may be suitable for reducing muscle fibrosis. These inhibitors block the TGF-β pathway by selectively binding to the TGF-βRI (Engebretsen et al., 2014; Suo et al., 2022; Peterson et al., 2022). Galunisertib, for example, has been shown to suppress the expression of collagen and alpha-smooth muscle actin (α-SMA) in human neonatal foreskin fibroblasts treated with TGF-β1 *in vitro* (Peterson et al., 2022). It has also passed phase II clinical trials for hepatocellular carcinoma and myelodysplastic syndrome, demonstrating a favorable toxicity profile (Fujiwara et al., 2015; Kelley et al., 2019). SM16 also inhibits collagen and α-SMA expression in TGF-β1-treated rat cardiac fibroblasts [16]. In addition, SM16 has been reported to prevent the induction of α-SMA-positive myofibroblasts and collagen production in a rat carotid injury model (Fu et al., 2008). Further exploring the therapeutic potential of these TGF-βRI inhibitors could open new avenues for enhancing healing in patients with muscle injuries, for example, after orofacial cleft surgery.

Human gingival fibroblasts show a consistent fibrotic response to TGF-β1 (Fadl and Leask, 2023). Additionally, C2C12 mouse myoblasts serve as a reliable model for studying myotube formation under controlled conditions (Allen et al., 2023). Therefore, this study investigates the effects of the TGF-βRI inhibitors AZ12799734, Galunisertib, and SM16, on myofibroblast differentiation from human gingival fibroblasts and myotube formation from C2C12 cells *in vitro*.

## 2 Materials and methods

### 2.1 The isolation of fibroblasts from human gingiva

Human gingival fibroblasts (HGFs) were isolated from gingival tissues obtained during third molar extractions, following written informed consent. The tissues were surgically collected and rinsed in phosphate-buffered saline (PBS; Gibco, Waltham, MA, United States) supplemented with 3% penicillin/streptomycin (Gibco) and 2% fungizone (Gibco). The gingival tissue was then cut into pieces (10 mm x 2–3 mm) and incubated in 5 mL of PBS containing 0.25% Dispase (Corning, Tewksbury, MA, United States) at 4°C overnight. The following day, the tissue pieces were washed with PBS and separated into epithelial and connective tissue layers. The connective tissue was minced and transferred to a 24-well plate containing 1 mL of culture medium. The culture medium consisted of Dulbecco’s Modified Eagle’s Medium (DMEM; Gibco) supplemented with 10% fetal bovine serum (FBS; Gibco) and 1% penicillin/streptomycin. The cells were then expanded and cryopreserved for future use. For this study, cells from passage 5 were utilized.

### 2.2 HGFs culture with TGF-β1 and TGF-βRI inhibitors

HGFs were seeded at a density of 1.5 × 10^3^ cells per well in 200 μL of culture medium in 96-well plates. The following day, the culture medium was replaced with fresh medium containing varying concentrations of human TGF-β1 (0, 1, 5, and 10 ng/mL; ImmunoTools, Friesoythe, Germany). TGF-β1-containing medium was refreshed every other day. On day 6, cells were washed with PBS, fixed in 4% formaldehyde in demineralized water for 10 min, and plates were stored at 4°C for subsequent immunofluorescence analysis. After determining the optimal concentration of TGF-β1, three different TGF-βRI inhibitors were tested at 0, 1, 5, 10, and 20 μM in the culture medium with TGF-β1. The selected concentrations were based on cytotoxicity data from our previous study (De Saeytyd et al., 2025). Briefly, we observed a dose-dependent decrease in cell viability only at higher concentrations. Galunisertib and AZ12799734 showed slight toxicity starting from 10 μM, whereas SM16 exhibited no significant cytotoxic effects. The TGF-βRI inhibitors were initially dissolved in DMSO and subsequently diluted in the culture medium, resulting in a final DMSO concentration of 0.1%. TGF-βRI inhibitors are AZ12799734 (4-[[4-[(2,6-Dimethyl-3-pyridinyl) oxy]-2-pyridinyl]amino]benzenesulfonamide, 4-[[4-[(2,6-Dimethylpyridin-3-yl) oxy]pyridin-2-yl]amino]benzenesulfonamide; Merck, Darmstadt, Germany), Galunisertib (LY2157299 monohydrate; Merck, Darmstadt, Germany) and SM16 (4-(5-(benzo[d][1,3]dioxol-5-yl)-4-(6-methylpyridin-2-yl)-1H-imidazole-2-yl) bicyclo [2.2.2]octane-1-carboxamide; MCE, Sollentuna, Sweden). The medium was replaced every other day, and on day 6, cells were fixed for immunofluorescence staining.

### 2.3 C2C12 culture with TGF-βRI inhibitors

C2C12 cells (ATCC, Virginia, United States) were cultured in the same medium as the HGFs, consisting of DMEM with 10% FBS and 1% penicillin/streptomycin. For differentiation, C2C12 cells were maintained in DMEM with 2% horse serum (Gibco) and 1% penicillin/streptomycin. Three different TGF-βRI inhibitors were each added at concentrations of 0, 1, 5, 10, and 20 μM in the differentiation medium.

C2C12 cells were seeded at a density of 6 × 10^3^ cells per well in 200 μL onto Matrigel-coated 96-well plates (CELLSTAR®, Greiner Bio-One, Alphen, NL). To prepare the Matrigel coating, Matrigel (Corning) was diluted 1:10 in DMEM, resulting in a final concentration of 1 mg/mL. Each well was coated with 20 μL of this solution and placed on ice for 10 min, after which the excess solution was removed. Plates were then left to dry for 2 h in a 37°C humidified cell culture incubator before cell seeding. The following day, the differentiation medium with different TGF-βRI inhibitors was added and replaced every other day. On day 6, cells were fixed for immunofluorescence analysis.

### 2.4 Immunofluorescence staining for myotubes and myofibroblasts

Fixed HGFs or C2C12 cell cultures in 96-well plates were washed with PBS and permeabilized with 0.5% Triton X-100 in PBS for 10 min. Cells were then incubated in a blocking buffer containing 1% normal goat serum, 10% w/v bovine serum albumin, 0.1% v/v Triton X-100, and 0.5% v/v Tween-20 in PBS for 1 h at room temperature. After washing with PBS, HGFs cultures were incubated with rabbit anti-α-SMA (1:250; AB_5694, Sigma, St. Louis, MO, United States), while C2C12 cultures were treated with mouse monoclonal anti-myosin heavy chain (MyHC) (the fast type II, 1:500; M4276, Sigma). The secondary antibody used was Alexa Fluor 647 goat anti-rabbit IgG (1:200; AB_2338580, Invitrogen, Waltham, United States) for HGFs cultures and Alexa Fluor 488 goat anti-mouse IgG (1:200; AB_2534069, Invitrogen) for C2C12 cultures. For nuclear visualization, DAPI (4′,6-diamidino-2-phenylindole, 0.4 μg/mL) in PBS was applied for 10 min, followed by rinsing with PBS and water. Finally, 20 μL of mounting medium containing 0.5% DABCO (1,4-diazabicyclo-(2,2,2) octane) in PBS (pH 8.6) was added to each well.

### 2.5 Quantification of immunostaining images

The image analysis was performed with ImageJ (Fiji, NIH-LOCI, Wisconsin, United States). Two Fiji macros were developed to count myofibroblasts and myotubes, respectively. The macro for myofibroblasts counts the number of nuclei within the α-SMA-stained areas, representing the number of myofibroblasts. In addition, it determines the total number of nuclei in the images. To validate this macro, we compared manual and automated myofibroblast counts on 16 random images. A Pearson correlation analysis revealed a correlation coefficient of 0.89 between manual and automated counts. Similarly, the macro for myotubes counts the number of MyHC-stained areas to determine the number of myotubes. Also, it counts the number of nuclei within myotubes and the total nuclei in the images. Validation of this macro using 16 random images, showed a correlation coefficient of 0.84 between manual and automated myotube counts. The high correlation values confirm the reliability of the automated macro counts. These macro sequences were automatically applied to all images in each experiment. The percentage of myofibroblasts was calculated by dividing the number of nuclei within the α-SMA-positive area by the total number of nuclei. Myoblasts’ fusion index (fusion efficiency) was determined by dividing the number of nuclei inside the myotubes by the total number of cells. The number of nuclei inside the myotubes divided by the stained myotube area indicates the size of myotubes.

### 2.6 RT-qPCR

Gene expression was analyzed using RT-qPCR. For the HGFs cultures, 4.5 × 10^4^ cells in 3 mL of culture medium were seeded per well onto non-coated 6-well plates. The culture medium with one of the three TGF-βRI inhibitors and TGF-β1 was replaced every other day. For the C2C12 cultures, 1.8 × 10^5^ cells in 3 mL of culture medium were seeded onto Matrigel-coated 6-well plates. From the next day, the differentiation medium with one of the three TGF-βRI inhibitors was replaced every other day. On day 3, after removing the culture medium and washing, the cells were harvested with Trizol (Invitrogen, Waltham, United States) followed by centrifuging at 400 g for 5 min at 4 C. The collected cell pellet was then used to isolate total RNA using a RNeasy Micro Kit (QIAGEN, Hilden, Germany) according to the manufacturer’s instructions. cDNA was made from the RNA using the iSCRIPT cDNA synthesis kit (BIO-RAD, CA, United States). RT-qPCR was performed using SYBR green supermix (BIO-RAD) with forward and reverse primers as shown in Table 1. The reaction conditions were 3 min at 95 C, followed by 39 cycles of 95 C for 15 s and 60 C for 30 s. The average Ct value of the experimental group and reference (GAPDH) genes were used to calculate ΔCt: ΔCt = Ct (target gene) – Ct (reference gene). The gene expression is represented in fold change compared to the reference gene (=2−ΔΔCT).

**TABLE 1:** Primer sequences for real-time PCR.

Gene	Forward primer	Reverse primer
*mGAPDH*	*GGCAAATTCAACGGCACA*	*GTTAGTGGGGTCTCGCTCCTG*
*mMyHC-II*	*CCGAGCAAGAGCTACTGGA*	*TGTTGATGAGGCTGGTGTTC*
*mMyoD*	*CCCCGGCGGCAGAATGGCTACG*	*GGTCTGGGTTCCCTGTTCTGTGT*
*mMyoG*	*ACTCCCTTACGTCCATCGTG*	*CAGGACAGCCCCACTTAAAA*
*hGAPDH*	*TGCACCACCAACTGCTTAGC*	*GGCATGGACTGTGGTCATGAG*
*hACTA2*	*GCTCACGGAGGCACCCCTGAA*	*TCCAGAGTCCAGCACGATG*
*hCOL1A1*	*CAGCCGCTTCACCTACAGC*	*TCAATCACTGTCTTGCCCCA*
*hKi-67*	*AAACCAACAAAGAGGAACACAAATT*	*GTCTGGAGCGCAGGGATATTC*

### 2.7 Statistics

All data passed the Shapiro-Wilk normality test and were analyzed using one-way ANOVA with a Tukey *post hoc* test, performed in GraphPad Prism version 9.00 (GraphPad, CA, United States). For all experiments, p-values of less than 0.05 were considered significant.

## 3 Results

### 3.1 TGF-β1 promotes HGF proliferation and myofibroblast differentiation

**FIGURE 1:**
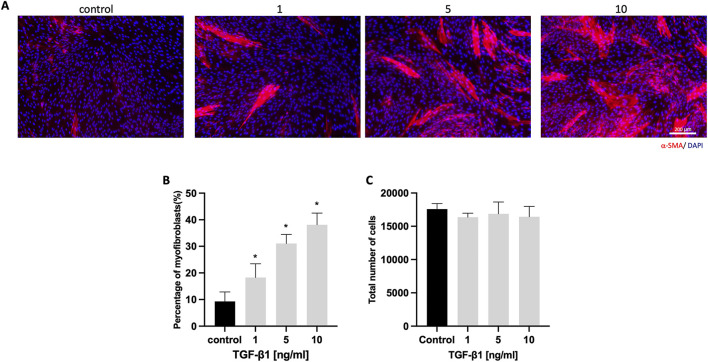
HGFs cultured with different concentrations of TGF-β1. **(A)** Representative immunofluorescence images of myofibroblasts with α-SMA (red) and DAPI (blue) staining after culturing HGFs without or with 1, 5, and 10 ng/mL TGF-β1 for 6 days; **(B)** Percentage of α-SMA-positive myofibroblasts with increasing concentrations of TGF-β1; **(C)** total cell number per well with increasing concentrations of TGF-β1. Scale bar: 200 µm. Statistical analysis was done with one-way ANOVA; *, indicates p < 0.05 (compared to control), N = 4.

HGFs were cultured with varying concentrations of TGF-β1 (0, 1, 5, and 10 ng/mL) for 6 days. Immunostaining revealed an increase in α-SMA-positive cells (Figure 1A). Quantitative analysis showed a significant rise in the percentage of myofibroblasts, from 9.3% ± 3.5% in the control to 38.1% ± 4.4% in the 10 ng/mL TGF-β1 group (Figure 1B). Additionally, TGF-β1 did not significantly affect total cell numbers across all concentrations (Figure 1C).

### 3.2 TGF-βRI inhibitors suppress myofibroblast differentiation

**FIGURE 2:**
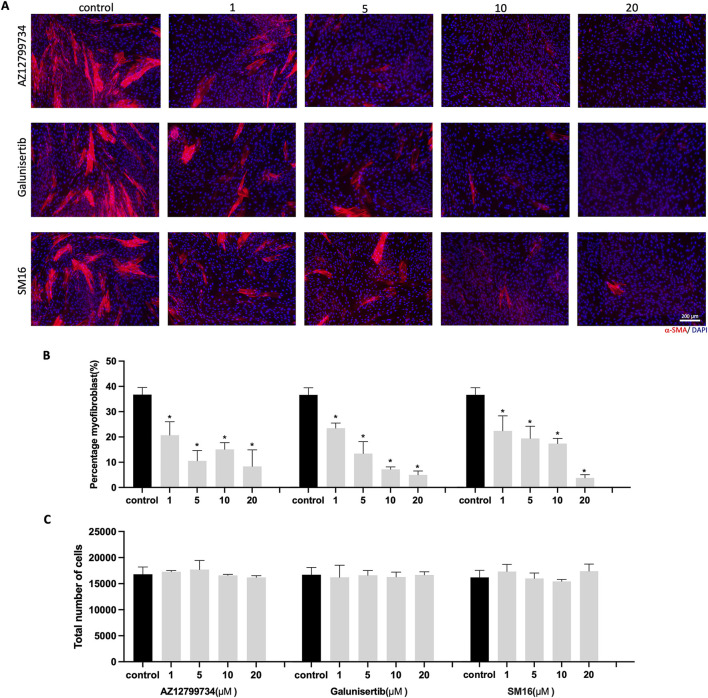
HGFs cultured with TGF-β1 and increasing concentrations of TGF-βRI inhibitors. **(A)** Representative immunofluorescence images of myofibroblasts with α-SMA (red) and DAPI (blue) staining after culturing HGFs with 10 ng/mL TGF-β1 and increasing concentrations of TGF-βRI inhibitors (AZ12799734, Galunisertib, and SM16) in culture medium with 0.1%DMSO and 10 ng/mL TGF-β1 for 6 days; **(B)** percentage of myofibroblasts with increasing concentrations of TGF-βRI inhibitors (with 0.1%DMSO and 10 ng/mL TGF-β1); **(C)** Total cell number per well with increasing concentrations of TGF-βRI inhibitors; Scale bar: 200 µm. Statistical analysis was done with one-way ANOVA; *, indicates p < 0.05 (compared to control), N = 4.

HGFs were cultured with 10 ng/mL TGF-β1 to mimic aspects of fibrosis. Varying concentrations of TGF-βRI inhibitors (0, 1, 5, 10, and 20 μM) were also added to the medium containing TGF-β1 for 6 days. Immunostaining showed a significant decrease in α-SMA-positive cells for all three TGF-βRI inhibitors (Figure 2A). Quantitative analysis confirmed the inhibition of myofibroblast differentiation, even at 1 μM, with reductions from 36.8% ± 2.8% in the control group to 20.7% ± 5.3%, 23.5% ± 2.1%, and 22.5% ± 6.0% for AZ12799734, Galunisertib, and SM16, respectively (Figure 2B). Total cell numbers did not significantly differ between groups (Figure 2C).

### 3.3 TGF-βRI inhibitors suppress myofibroblast expression of fibrotic genes and a proliferation marker

**FIGURE 3:**
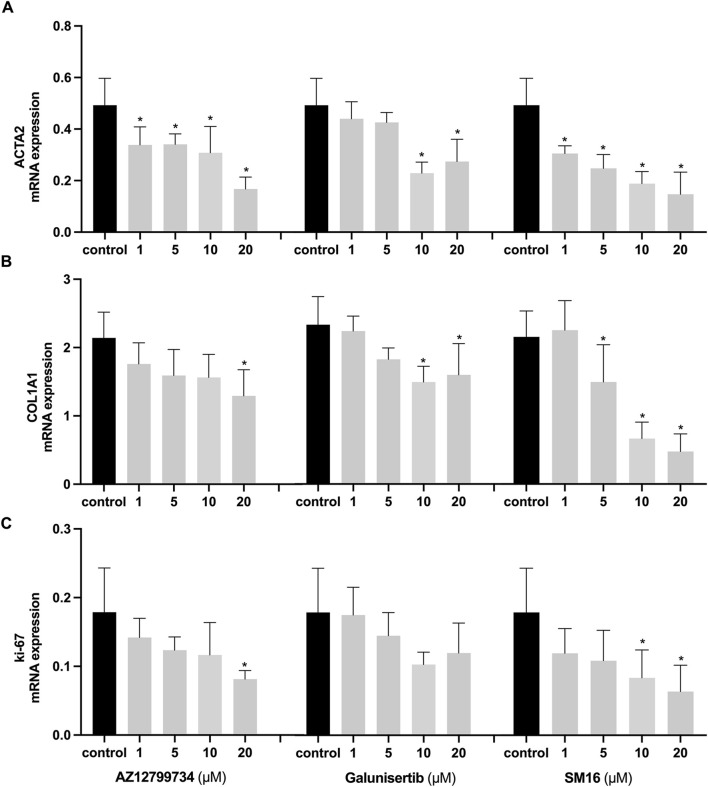
Effects of TGF-βRI inhibitors on gene expression. Expression analyses of ACTA2 **(A)**, COL1A1 **(B)**, and Ki-67 **(C)** after culturing HGFs with increasing concentrations of the TGF-βRI inhibitors (AZ12799734, Galunisertib, and SM16) in culture medium with 0.1%DMSO and 10 ng/mL TGF-β1 for 3 days in the presence of 10 ng/mL TGF-β1. The reference gene was GAPDH. Statistical analysis was done with one-way ANOVA; *, indicates p < 0.05 (compared to control), N = 4.

We further assessed the impact of the three TGF-βRI inhibitors on gene expression linked to myofibroblast differentiation and HGF proliferation. Consistent with α-SMA immunostaining results, ACTA2 expression was significantly inhibited by all three TGF-βRI inhibitors, even at the lowest concentration (Figure 3A). Collagen type I alpha 1 chain (COL1A1) exhibited reduced expression with all three inhibitors (Figure 3B), while the proliferation marker Ki-67 decreased only after treatment with SM16 and AZ12799734 (Figure 3C).

### 3.4 Effects of TGF-βRI inhibitors on myotube formation

**FIGURE 4:**
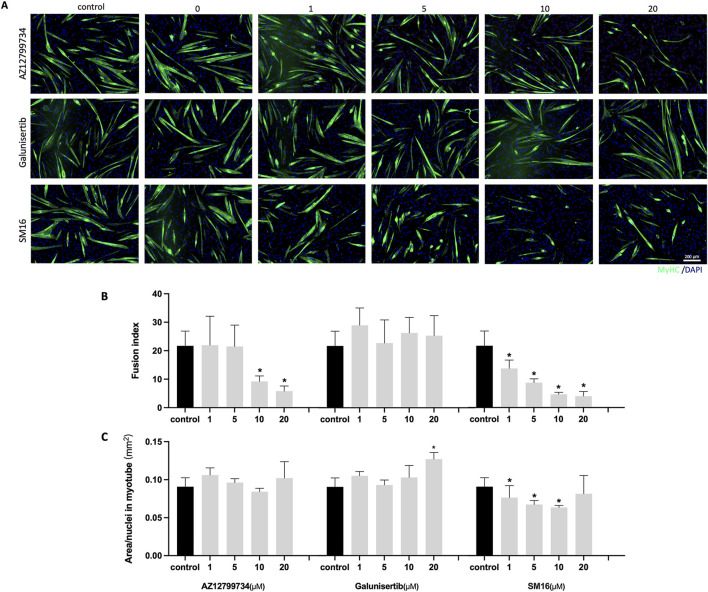
Myotube formation after TGF-βRI inhibitors exposure. **(A)** Representative immunofluorescence images of myotubes stained for MyHC (green) and DAPI (blue) staining after culturing of C2C12 cells with increasing concentrations of TGF-βRI inhibitors (AZ12799734, Galunisertib, and SM16) in differentiation medium with 0.1%DMSO for 6 days; **(B)** Fusion index with increasing concentrations of TGF-βRI inhibitors; **(C)** Myotube area divided by the number of nuclei in myotubes with increasing concentrations of TGF-βRI inhibitors. Scale bar: 200 µm. Statistical analysis was done with one-way ANOVA; *, indicates p < 0.05 (compared to control), N = 4.

C2C12 cells were cultured with increasing concentrations of the three TGF-βRI inhibitors (0, 1, 5, 10 and 20 μM) in differentiation medium for 6 days. Immunostaining indicated a reduction in the number of myotubes with AZ12799734 and SM16. In contrast, Galunisertib showed no obvious effect on myotube formation (Figure 4A). Quantitative analysis demonstrated a significant decrease in fusion index for AZ12799734 (5.8% ± 1.8%) and SM16 (4.0% ± 1.6%), compared to the control (21.7% ± 5.2%) at 20 μM, with no differences for Galunisertib (Figure 4B). Quantification of myotube size showed no differences between AZ12799734, SM16, and the control group (Figure 4C). Interestingly, Galunisertib slightly increased myotube size to 0.13 ± 0.01 mm^2^/nucleus from 0.09 ± 0.01 in the control group (p < 0.05) (Figure 4C).

### 3.5 Effects of TGF-βRI inhibitors on the expression of myoblast differentiation markers

**FIGURE 5:**
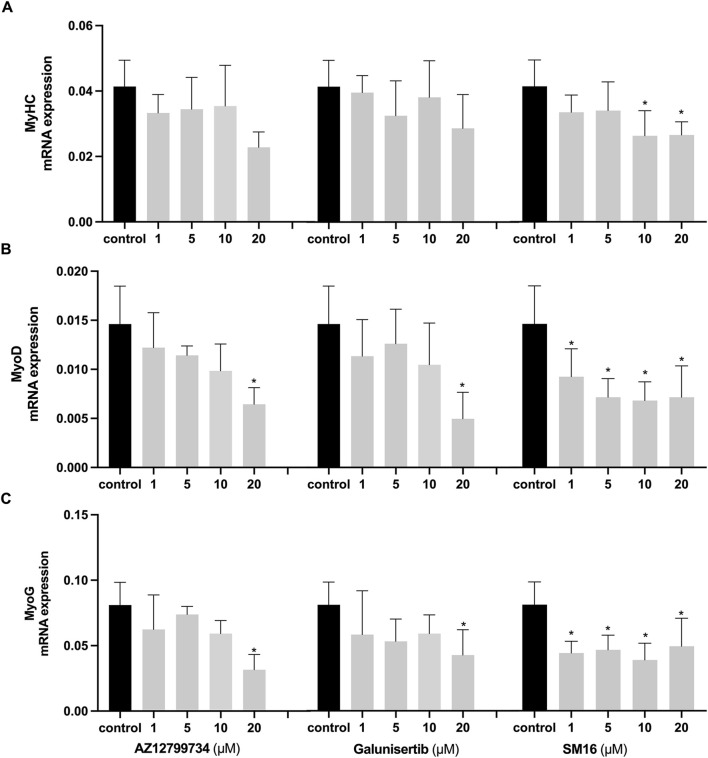
Effects of TGF-βRI inhibitors on the expression of myoblast differentiation markers Expression analyses of myoblast differentiation markers, MyHC **(A)**, MyoD **(B)** and MyoG **(C)** after C2C12 cells cultured with increasing concentrations of TGF-βRI inhibitors (AZ12799734, Galunisertib, and SM16) in differentiation medium with 0.1%DMSO for 3 days. The reference gene for PCR was GAPDH. Statistical analysis was done with one-way ANOVA; *, indicates p < 0.05 (compared to control), N = 4.

Expression analyses further demonstrated the effects of the inhibitors on myoblast differentiation markers. MyHC expression did not significantly differ between the Galunisertib-treated and the control group, although at 20 μM, Galunisertib inhibited the expression of myoblast determination protein 1 (MyoD) and myogenin (MyoG) (Figure 5). Consistent with its effect on the fusion index, AZ12799734 significantly inhibited MyHC, MyoD, and MyoG expression in the highest concentration, while SM16 significantly inhibited MyoD and MyoG expression even at lower concentrations.

## 4 Discussion

For Muscle regeneration after surgery or trauma is often hindered by fibrosis, resulting in both functional and aesthetic complications. Antifibrotic therapies generally aim to inhibit the differentiation of myofibroblasts, the key cells in fibrosis (Henderson, Rieder, and Wynn, 2020). TGF-β1 plays a pivotal role in fibrosis, making it a key target for fibrosis treatment (Bersini et al., 2018). Our data demonstrate that TGF-β1 induces myofibroblast differentiation as in previous studies (Qu et al., 2019; Meran et al., 2007; Bell et al., 2022; Kottmann et al., 2012). AZ12799734, Galunisertib, and SM16, three TGF-βRI inhibitors, have not been studied in the context of muscle fibrosis and may hold potential for treatment. Thus, we examined their effects on myofibroblast differentiation and myotube formation to explore their therapeutic potential.

We first evaluated the effects of AZ12799734, Galunisertib, and SM16 on myofibroblast differentiation from HGFs cultured in the presence of TGF-β1. TGF-β1 was added to the culture medium to mimic aspects of fibrosis, consistent with previous studies (Cheng et al., 2020; Yang et al., 2022; Joannes et al., 2023). As expected, our findings show that all three TGF-βRI inhibitors reduced myofibroblast differentiation in HGFs treated with TGF-β1. They also decreased the deposition of extracellular matrix (ECM) components, as evidenced by reduced COL1A1 gene expression. Previous studies have also reported that SM16 decreases myofibroblast differentiation and COL1A2 expression in rat cardiac fibroblasts treated with TGF-β1, while Galunisertib reduces COL1A1 and α-SMA expression in TGF-β1-treated human dermal and renal fibroblasts (Bigaeva et al., 2020; Peterson et al., 2022). *In vivo* studies have demonstrated that SM16 significantly reduces COL1A2 expression and ECM production in a mouse model of cardiac fibrosis induced by pressure overload, thereby mitigating fibrotic progression (Bjørnstad et al., 2012; Engebretsen et al., 2014). Similarly, localized application of Galunisertib via a patch in a rat model of myocardial infarction effectively inhibited α-SMA expression and effectively attenuated myocardial fibrosis (H. Chen et al., 2022). Although no previous studies have reported the effects of AZ12799734 on fibrosis and COL1A1 and α-SMA expression, similar effects are expected, as the mechanism of action is similar to that of the other two (Goldberg et al., 2009). Thus, these findings support the potential of these three inhibitors in suppressing TGF-β-induced myofibroblast differentiation and collagen formation.

Our findings also show that only SM16 and AZ12799734 significantly inhibited Ki-67 expression. By contrast, the total cell number remained unchanged. Ki-67 interacts with the nucleolus to regulate cell proliferation by participating in ribosomal RNA transcription during the G1 and S phases (Booth et al., 2014). The variation in our results may be due to differences in the timing of Ki-67 expression analysis and cell counting. Ki-67 expression was assessed on day 3, indicating that SM16 and AZ12799734 inhibited proliferation. However, by day 6, all cultures had probably already reached confluency, despite the earlier differences in proliferation.

We next examined the effects of the TGF-βRI inhibitors on myotube formation in C2C12 cells. Both AZ12799734 and SM16 inhibited myotube formation, while Galunisertib had no reducing effect, but even slightly increased myotube size. Currently, no other studies have focused on the impact of these three TGF-βRI inhibitors on myotube formation. However, one study reported that inhibition of TGF-βRI by SB 431542, another specific TGF-βRI inhibitor, promoted both myosin expression and fusion in C2C12 cells (Droguett et al., 2010). In our study, AZ12799734, Galunisertib, and SM16 exhibit varying effects on myotube formation, while similarly inhibiting myofibroblast differentiation. This may be caused by slight variations in their mechanisms of action, as discussed below.

AZ12799734, Galunisertib, and SM16 are all small-molecule inhibitors of TGF-βRI with a comparable mechanism of action (Hill, 2016). The heteromeric receptor complex of TGF-βRI and TGF-βRII, activated by TGF-β, triggers diverse downstream signaling cascades to modulate gene expression (Peng et al., 2022). Among the activin receptor-like kinases (ALK1–7) within the TGF-βRI family, ALK4, ALK5, and ALK7 activate the transcriptional coregulators Smad2 and Smad3, while ALK1, ALK2, ALK3, and ALK6 activate Smad1, Smad5, and Smad8. ALK5 is central to the canonical TGF-β/Smad pathway, where TGF-β binding triggers the phosphorylation of Smad2/3, their nuclear translocation, and subsequent transcriptional activation. Although non-canonical Smad-independent pathways, such as MAP kinase (MAPK) pathways (ERK, JNK, P38), Rho-like GTPase signaling pathways, and PI3K/AKT pathways, are not direct downstream components of ALK5 signaling, they might interact with this pathway (Pannu et al., 2007; Zhang, 2009).

Despite all being TGF-βRI inhibitors, competitive binding assays show that AZ12799734, Galunisertib, and SM16 have distinct binding affinities for ALK5, with affinity constants (Kd) of 6.6 nM (Fu et al., 2008), 52 nM (Tauriello et al., 2024), and 740 nM (Spender et al., 2019), respectively. Additionally, they demonstrate varying half-maximal inhibitory concentrations (IC_50_) when occupying the ATP-binding site of ALK5. For instance, SM16 inhibits ALK5 activity in HepG2 cells with an IC_50_ of 64 nM (Suzuki et al., 2007), while Galunisertib exhibits an IC_50_ of 380 nM in NIH3T3 cells (Fu et al., 2008). Therefore, AZ12799734, Galunisertib, and SM16 can bind to ALK5 with varying binding affinities and strengths, which mainly regulate canonical signaling. Given the considerable homology among the ATP-binding sites of ALK kinases, Galunisertib also exhibits weak inhibitory activity against ALK3/BMPR1A (IC_50_ = 16.8 μM) in HEK293 cells (Yingling et al., 2017), while SM16 inhibits p38/SAPK2a in NIH3T3 cells with an IC_50_ of 0.8 μM (Fu et al., 2008). In conclusion, AZ12799734, Galunisertib, and SM16 display varying binding affinities and inhibition strengths across multiple ALK kinases associated with both canonical and non-canonical pathways. This may be related to their differing effects observed in our study.

AZ12799734, Galunisertib, and SM16 all suppress α-SMA and COL1A1 expression by inhibiting the Smad2/3-dependent pathway (Luangmonkong et al., 2017; Mansour et al., 2024; Leeuwen et al., 2024). Silencing Smad2/3 using specific siRNAs increases MyoG expression and enhances myotube formation in C2C12 cells (Droguett et al., 2010). This suggests that these inhibitors inhibit myofibroblast differentiation and simultaneously promote myotube formation via the canonical pathway. However, their cellular effects may vary due to differences in binding affinity or receptor interactions as discussed above, but they might also differentially activate non-canonical pathways. SM16 exhibits weak inhibitory activity against p38 kinases as shown by kinase selectivity assays (Fu et al., 2008). In addition, AZ12799734 inhibits Smad1 phosphorylation by binding to ALK1/2, leading to off-target inhibition of bone morphogenetic protein (BMP) receptors (Chen et al., 2023; Spender et al., 2019). Inhibition of p38 signaling by SM16 has been reported to reduce fibrosis in a rat model of carotid injury (Fu et al., 2008), while activation of BMP signaling can enhance TGF-β signaling and promote fibrosis in cancer (Docshin et al., 2024). This suggests that inhibition of these non-canonical pathways may also contribute to the antifibrotic effects The p38, MAPK and BMP/Smad signaling pathways are also known to positively influence muscle cell differentiation, including that of satellite cells (SCs) and C2C12 myoblasts, as previously reviewed (Brennan et al., 2021; Sartori, Gregorevic, and Sandri, 2014). Activating p38 with creatine enhances myotube formation in C2C12 cells, whereas inhibition with SB203580 reduces myotube formation in SCs (Kim et al., 2023; Deldicque et al., 2007). Additionally, BMP4 promotes muscle growth in ΔIg3-MuSK mice by inducing Smad1/5/8 phosphorylation via ALK1, ALK2, and ALK3 receptors (Yilmaz et al., 2016; Jaime et al., 2024). Thus, SM16 and AZ12799734 may also impair myotube formation by suppressing the non-canonical pathways.

This study is the first to investigate the *in vitro* effects of AZ12799734, Galunisertib, and SM16 on both myofibroblast differentiation and myotube formation. Our findings suggest that Galunisertib is the most promising candidate for further research on treating muscle fibrosis. Furthermore, Galunisertib has a high hydrophobicity, facilitating efficient translocation across cell membranes, making it suitable for formulation as a topical cream for localized drug delivery (Peterson et al., 2022). Although concerns have been raised regarding the potential toxicity of ALK5 inhibitors, several *in vivo* studies have reported effective tumor suppression or antifibrotic outcomes without significant side effects (Jing et al., 2025; Sounbuli et al., 2022; Tschernia and Gulley, 2022), Specifically, Galunisertib has shown a favorable safety profile, with no significant toxicity observed in preclinical models, for instance, in a murine model of breast cancer (Yingling et al., 2017), as well as in bone marrow fibrosis (Yue et al., 2015) and liver fibrosis models (Hammad et al., 2018). Moreover, many clinical studies have explored the therapeutic potential of TGF-βRI inhibitors. Notably, no significant adverse effects have been reported for Galunisertib in human clinical studies employing intermittent dosing regimens (Gulley et al., 2022; Herbertz et al., 2015). Currently (13 February 2025), 19 completed, 3 ongoing, and 1 terminated clinical trials have been performed with Galunisertib (clinicaltrials.gov). The ongoing trials focus on conditions such as colorectal cancer with peritoneal metastases (NCT05700656), prostate cancer (NCT02452008), and rectal cancer (NCT02688712). However, it is crucial to recognize that muscle fibrosis and regeneration are complex, long-term processes that encompass more than just the early cellular and molecular events explored in this study. Our current focus has primarily been on early-stage markers, such as myofibroblast differentiation and the myotube fusion index. To gain deeper insights into the mechanisms and therapeutic potential of Galunisertib, further RNA-seq analyses of human SCs and comprehensive *in vivo* studies are necessary. These future investigations should aim to evaluate its efficacy in promoting myofiber maturation and resolving established fibrosis.

## Data Availability

The original contributions presented in the study are included in the article/Supplementary Material, further inquiries can be directed to the corresponding author.

## Supplementary material

SUPPLEMENTARY VIDEO S1 Spontaneous twitching of differentiated C2C12 myotubes after 6 days of culture. C2C12 myoblasts were cultured in differentiation medium for 6 days to induce myotube formation. The video demonstrates spontaneous contractions, indicating functional maturation and myogenic differentiation of the myotubes.

## Abbreviations

TGF-β: Transforming growth factor beta; TGF-βRI: TGF-β receptor I; MyHC: Myosin Heavy Chain; MyoD: Myoblast determination protein 1; MyoG: Myogenin; ECM: Extracellular matrix; SCs: Satellite cells; α-SMA: Alpha smooth muscle actin; IPF: Idiopathic pulmonary fibrosis; ALK5: activin receptor-like kinase 5; COL1A1: Collagen Type I Alpha 1 Chain.
